# Analysis of HPV prevalence among individuals with reproductive tract infections in a Chinese population

**DOI:** 10.1097/MD.0000000000034989

**Published:** 2023-10-13

**Authors:** Yu-Xia Zhou, Liang Wang, Ting-Ting Wang, Xiao-Li Qu, Xiao-Qian Zhang

**Affiliations:** a Key Laboratory of Birth Regulation and Control Technology of National Health Commission of China, Maternal and Child Health Care Hospital affiliated to Qingdao University, Jinan, China; b Research Center of Traditional Chinese Medicine and Clinical Pharmacy, Maternal and Child Health Care Hospital Affiliated to Qingdao University, Jinan, China.

**Keywords:** age, bacterial vaginitis, fungal vaginitis, HPV

## Abstract

The previous research has found that human papillomavirus (HPV) infection is the main cause of cervical cancer, but it is still unclear whether HPV infection, as well as the HPV genotypes, are related to reproductive tract infections in the Chinese population. Patients who underwent HPV screening at Shandong Maternal and Child Health Hospital were selected, and the HPV infection status was analyzed among patients with cervical lesions, bacterial vaginosis, cervical inflammation, fungal vaginitis, and pelvic infections. SPSS 22 statistical analysis was used to analyze the differences in HPV infection types and rates between the control group and the experimental group. The HPV infection rate of bacterial vaginosis (χ^2^ = 13.4; *P* < .001) and fungal vaginitis (χ^2^ = 3.3; *P* < .045) are both significantly different from the control group. The single HPV infections reveals significant differences from control group in bacterial vaginosis (χ^2^ = 7.3; *P* = .004), fungal vaginitis (χ^2^ = 4.5; *P* = .023), and cervical lesions (χ^2^ = 58.8; *P* < .001). In the bacterial infection group, HPV51 (1.9%; χ^2^ = 6.0; *P* = .008) and HPV58 (4.7%; χ^2^ = 3.3; *P* = .044) showed significant differences in infection compared to the control group. In the fungal infection group, HPV39 (2.7%; χ^2^ = 4.7; *P* = .032) showed a significant difference in infection compared to the control group. Cervical lesions, bacterial vaginosis, fungal vaginitis, and cervical lesions among Chinese population exhibit age-specified distribution. HPV infection rate in bacterial vaginitis, fungal vaginitis and cervical lesions was higher than that in normal group. HPV52 and HPV16 infection are different, and HPV39 is different between bacterial vaginitis and fungal vaginitis.

## 1. Introduction

Human papillomavirus (HPV) is one of the most widely transmission DNA viruses worldwide, identified as prime culprit of cervical cancer.^[1,2]^ Despite the important role of HPV vaccination in preventing cervical cancer, it is unclear the harmful of HPV infection to reproductive tract infections.^[3]^ Previous studies have shown that HPV infection is associated with vaginal microbial imbalance, also, HPV infection changed the richness and diversity of the vaginal microbiota.^[4,5]^ At same time, How vaginal microecology and HPV co-infection to affect human health need to be addressed.

Previous studies have shown that HPV infection is associated with vaginal microbial imbalance.^[6,7]^ Pelvic inflammatory disease is an inflammation of the fallopian tubes and cervix, caused by microecological imbalance in the reproductive tract triggered by pathogenic microorganisms including *Neisseria gonorrhea, Chlamydia trachomatis*, and *Mycoplasma genitalium*.^[8]^ Bacterial vaginitis is one of the most common vaginal microecological imbalance, caused by increasing in *Gardnerella vaginalis, Prevotella, Atopobium vaginalis, Bacteroidetes, Streptococcus*, and a decrease in the normal flora Lactobacillus.^[9,10]^ Studies have shown that more than 75% of women have got vaginal candida and that *Candida albicans* is the main causative agent of fungal vaginitis.^[11]^ In conclusion, alterations in the microecology of the female genital tract are closely related to changes in HPV invasiveness.^[12]^

More than 200 HPV genotype have been identified, and HPV genotype show different preference and specified in invading host.^[13]^ For example, the main HPV genotype causing cervical cancer are HPV18 and HPV52, while the main HPV genotype causing squamous cell carcinoma is HPV16.^[14,15]^ Few studies have been carried out on invading bias of HPV genotype in genital tract microenvironment. Previous studies have shown that HPV58 and HPV52 is prone to invading the women got bacterial vaginitis in Northwest China, while further research on the relationship between HPV genotype with microecological dysregulation of the reproductive tract is required.^[16]^

HPV transmission shows significant variability among ethnic, regional, and temporal.^[17,18]^ Jinan, as the capital city of Shandong Province with a population size of more than 10 million, is a representative geographic area for epidemiologic investigations in East China. In this study, we investigated the subtypes of HPV infection under different vaginal microecological types in Shandong Province of China, to provide new knowledge of the dangers of reproductive tract microecological imbalance and HPV prevention.

## 2. Methods

### 2.1. Ethics statement

The research was approved by the Ethics Committee of Shandong Maternal and Child Health Hospital (No. 2021-024), in accordance with the China Ethical Principles for Biomedical Research Involving Human Subjects and the Declaration of Helsinki for Human Research. The content of the experiment (including the use of tissue samples), the significance of research, and confidentiality of the study have been well informed to the participants, the research was well-informed and supported by all subjects and/or their legal guardian(s) in case of minors.

### 2.2. Study population and information collection

#### 2.2.1. Data collection.

The data were collected from individuals who sought medical advice in Shandong Maternal and Child Health Hospital from January 2018 to August 2021. A total of 13,972 individuals were enrolled in this study. The enrolled individuals were aged from 15 to 83 years.

There are separately 239,659,294,943 individuals enrolled in pelvic inflammatory, cervical lesions, fungal vaginitis, or bacterial vaginitis.

Inclusion criteria for the pelvic inflammatory group: pressure pain in the lower abdomen and adnexal area; purulent discharge of cervical or vaginal mucus or the presence of leukocytes on microscopic examination of vaginal secretions; accelerated blood sedimentation or elevated levels of reactive protein.

Inclusion criteria for cervical lesions: liquid-based thin-layer cytology examination and colposcopic histopathological diagnosis, cervical intraepithelial low-grade tumor-like lesions.

Inclusion criteria for bacterial vaginitis: a. positive clue cells (>20%). b. positive ammonia test. c. vaginal pH > 4.5.

Inclusion criteria for fungal vaginitis: a. wet film microscopy of vaginal secretions reveals budding spores, pseudomycorrhizae or mycelium. b. Fungal culture of vaginal secretions or commercial tests showing positive for Pseudomycetes.

The control population group was the physical control group of the physical examination population. There are no gynecological diseases mentioned above among healthy individuals.

No enrolled individuals with a basic disease (e.g., hypertension, diabetes, dyslipidemia, chronic bronchitis, chronic obstructive pulmonary disease, chronic gastropathy, hereditary diseases, cardiovascular and cerebrovascular diseases, etc) were included.

#### 2.2.2. Sample size estimation.

According to the formula N = 2pq[(Z_α_+Z_β_)/(p1−p2)]^2^, assuming the same sample size for the experimental and control groups, α is taken as 0.05, Z value is two-sided, Z_α_ = 1.96, β test efficacy is 0.8, Z_β_ = 0.84; p1 = 0.12, p2 = 0.22, N ≈ 221, so N > 221.

#### 2.2.3. HPV genotyping.

HPV detection and genotyping were performed using an HPV Genotyping Test Kit (Life River Co., Shanghai, China). The kit was used to detect and genotype fifteen high-risk HPV genotypes (HPV16, HPV18, HPV31, HPV33, HPV35, HPV39, HPV45, HPV51, HPV52, HPV56, HPV58, HPV59, HPV66, HPV68, and HPV82) and two low-risk HPV genotypes (HPV6/HPV11). The experimental steps followed the kit instructions (Life River Co., Shanghai, China).

#### 2.2.4. HPV type-specific prevalence.

The positive individuals were divided into single HPV genotype, double HPV genotype and triple genotype according to the infection genotype. The data were statistically analyzed using SPSS 22.0 software (SPSS lnc., Chicago, IL). Any differences between the groups in prevalence were assessed by chi-squared (χ2) tests, and *P* < .05 was considered statistically significant.

#### 2.2.5. Age-specific disease.

The statistical sample was divided into 9 age groups, namely, <20 years group, 21 to 25 years group, 26 to 30 years group, 31 to 35 years group, 36 to 40 years group, 41 to 50 years group, 51 to 60 years group, and > 60 years group. The HPV infection prevalence in each group was presented using line chart.

## 3. Results

### 3.1. The prevalence of HPV infection rate among individuals overall

This study presents 2828 positive from 13,972 individuals, and the positive rate was 20.3%. The HPV single genotype group had 2100 cases, accounting for 74.3% of the positive individuals; the HPV double genotype group had 520 cases, accounting for 18.4% of the positive individuals; and the HPV triple genotype group had 208 cases, accounting for 7.4% of the positive individuals.

In the statistics, the top fifth prevalent HPV genotypes were HPV52 (5.1% of the total individuals), HPV16 (3.9%), HPV58 (3.2%), HPV51 (2.2%), and HPV39 (1.9%). The top fifth single infection types were HPV52 (21.0% of the HPV single type individuals), HPV16 (15.9%), HPV58 (12.6%), HPV51 (8.6%), and HPV39 (7.1%); The top fifth double infection types were HPV52 (31.7% of the HPV double types individuals), HPV16 (27.5%), HPV58 (21.7%), HPV51 (15.4%), and HPV39 (15.2%); the top fifth triple infection types were HPV52 (65.4% of the HPV triple types individuals), HPV16 (43.6%), HPV58 (43.6%), HPV51 (35.9%), and HPV39 (35.9%) (Table 1).

**Table 1:** Prevalence of the different HPV genotypes among the included individuals.

Genotypes	Sorts				
	HPV positive (ratio)	Single type (ratio)	Double types (ratio)	Triple types (ratio)	Total
HPV 16	542 (19.7%)	331 (15.9%)	143 (27.5%)	68 (43.6%)	13,933
HPV 18	192 (7.0%)	115 (5.5%)	48 (9.2%)	29 (18.6%)	13,933
HPV 31	131 (4.8%)	67 (3.2%)	41 (7.9%)	23 (14.7%)	13,933
HPV 33	117 (4.2%)	54 (2.6%)	37 (7.1%)	26 (16.7%)	13,933
HPV 35	92 (3.3%)	36 (1.7%)	29 (5.6%)	27 (17.3%)	13,933
HPV 39	266 (9.6%)	148 (7.1%)	79 (15.2%)	39 (25.0%)	13,933
HPV 45	57 (2.07%)	18 (0.9%)	23 (4.4%)	16 (10.3%)	13,933
HPV 51	314 (11.4%)	178 (8.6%)	80 (15.4%)	56 (35.9%)	13,933
HPV 52	704 (25.5%)	437 (21.0%)	165 (31.7%)	102 (65.4%)	13,933
HPV 56	220 (8.0%)	106 (5.1%)	71 (13.7%)	43 (27.6%)	13,933
HPV 58	443 (16.1%)	262 (12.6%)	113 (21.7%)	68 (43.6%)	13,933
HPV 59	170 (6.2%)	83 (4.0%)	40 (7.7%)	47 (30.1%)	13,933
HPV 66	235 (8.5%)	120 (5.8%)	65 (12.5%)	50 (32.05%)	13,933
HPV 68	198 (7.2%)	92 (4.4%)	63 (12.1%)	43 (27.6%)	13,933
HPV 82	70 (2.5%)	27 (1.3%)	28 (5.4%)	15 (9.6%)	13,933
HPV6/11	79 (2.8%)	35 (1.7%)	23 (4.4%)	21 (10.1%)	2573
Total	2828	2100	520	208	13,972

The ratio is calculated by the HPV-genotypes positive individual divided the total HPV positive.

### 3.2. Age description of 5 reproductive tract infections included in the study

**Figure 1.**
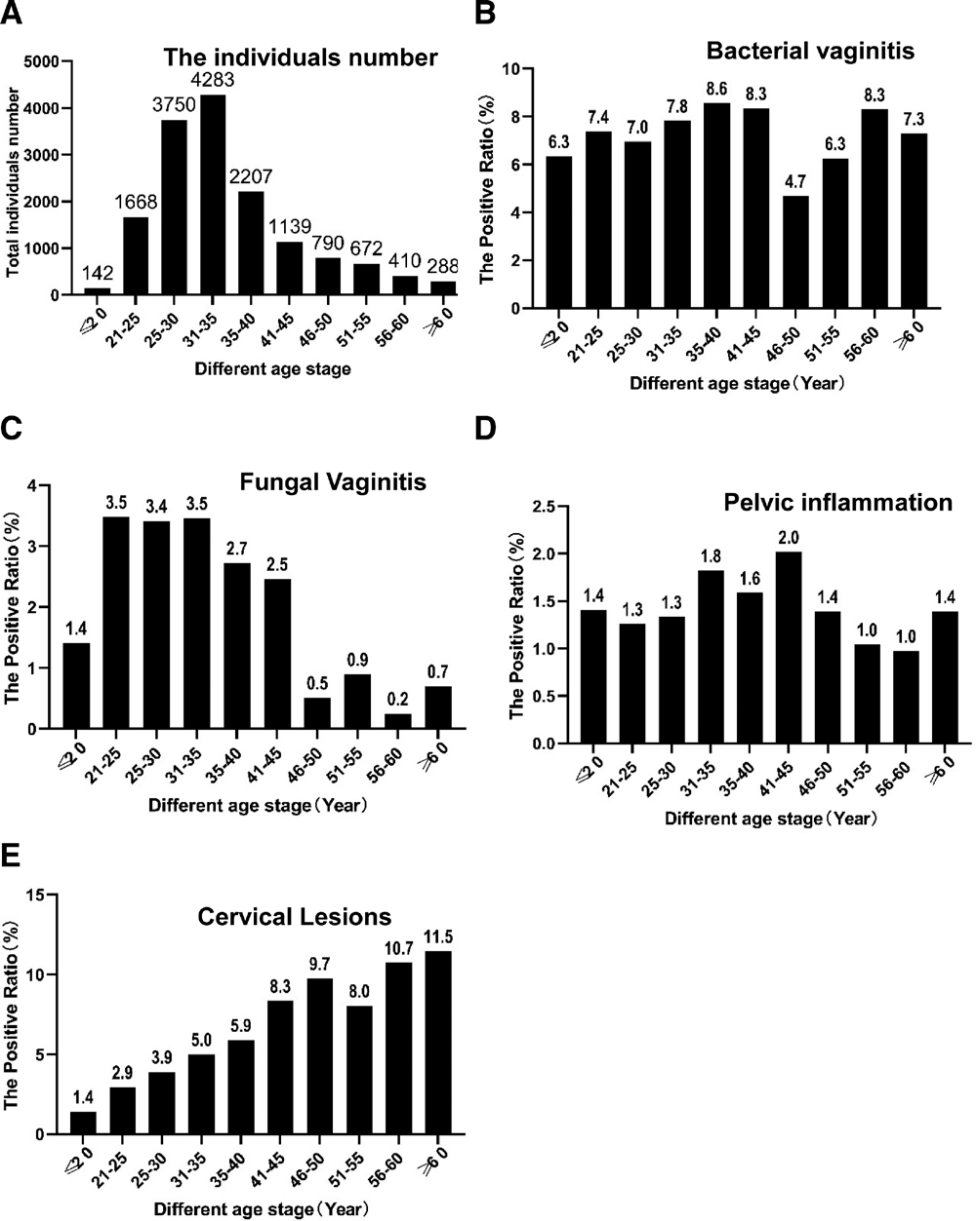
Age-specified infection ratios of fungal vaginitis, bacterial vaginitis, cervical lesions, and pelvic inflammatory. (A) The total number enrolled at different stage. (B–E) Represent the positive ratio of bacterial vaginitis, fungal vaginitis, inflammatory disease and cervical lesions respectively. The ratio is calculated by positive individuals/total individuals.

The age distribution of individuals with reproductive tract infections in the study population is mostly between 25 to 40 years old, with the highest population in the 31 to 35 age group. The incidence of bacterial vaginosis increases with age, reaching its highest infection rate between 35 to 45 years old, followed by a slight decrease in infection rate. The incidence of fungal vaginitis is higher between 21 to 35 years old, and then decreases significantly. Patients with cervicitis have a high incidence between 25 to 45 years old, with a decrease in incidence after 45 years old. The age group with the highest incidence of pelvic inflammatory disease is between 41 to 45 years old, with a significant decrease in incidence after 45 years old. Interestingly, the incidence of cervical lesions increases with age (Fig. 1).

### 3.3. Comparison of overall HPV infection rates among the 5 reproductive tract infections

The infection rate of bacterial vaginosis is 22.9%, which is significantly different from the control group in terms of HPV infection (χ^2^ = 13.4; *P* < .001). The infection rate of fungal vaginitis is 18.8%, which also shows a significant difference compared to the control group (χ^2^ = 3.3; *P* < .045). The overall infection rate of cervical lesions is 40.1%, which is significantly different from the control group (χ^2^ = 3.3; *P* < .001) (Table 2). Analysis of single HPV infections reveals significant differences compared to the control group in bacterial vaginosis (χ^2^ = 7.3; *P* = .004), fungal vaginitis (χ^2^ = 4.5; *P* = .023), and cervical lesions (χ^2^ = 58.8; *P* < .001). Analysis of double HPV infections shows significant differences compared to the control group in bacterial vaginosis (χ^2^ = 4.8; *P* = .017) and cervical lesions (χ^2^ = 11.3; *P* < .001) (Table 3).

**Table 2:** Prevalence of the HPV^+^ among the individuals with disease.

Disease	HPV^+^							
	HPV^+^		Single HPV^+^		Double HPV^+^		Triple HPV^+^	
	No.	Ratio	No.	Ratio	No.	Ratio	No.	Ratio
Control	61	14.3%	44	10.3%	10	2.3%	7	1.6%
Pelvic	45	18.8%	30	12.6%	8	3.3%	7	2.9%
CL	264	40.1%	199	30.2%	46	7.0%	19	2.9%
FV	57	18.8%	46	15.1%	4	1.3%	7	2.3%
BV	216	22.9%	149	15.8%	47	4.9%	21	2.2%

The ratio is calculated by the total HPV positive, the single HPV positive, double HPV positive or triple HPV positive individuals, divided the total infected individuals respectively.

BV = bacterial vaginitis, CL= cervical lesions, FV=fungal vaginitis, No. = sample number.

**Table 3:** Chi-square test for differences in HPV positivity among genital tract infection groups.

Genotypes	Sorts							
	HPV^+^		Single HPV^+^		Double HPV^+^		Triple HPV^+^	
	χ^2^	*P*	χ^2^	*P*	χ^2^	*P*	χ^2^	*P*
Pelvic	2.3	.080	0.8	.227	0.6	.299	1.2	.202
CL	81.7	.001	58.8	.001	11.3	.001	1.7	.135
FV	3.3	.045	4.5	.023	0.9	.256	0.5	.329
BV	13.4	.001	7.3	.004	4.8	.017	0.5	.316

All 4 groups were compared with the health check group.

BV = bacterial vaginitis, CL = cervical lesions, FV = fungal vaginitis.

### 3.4. Comparison of positivity rates for the top 6 HPV subtypes among the reproductive tract infections

In the analysis of the most prevalent HPV subtypes in the reproductive tract, the main HPV subtypes in each group are the same as those in the control group. The infection rates of the top 6 types were compared between the control and experimental groups. In the bacterial infection group, HPV51 (1.9%; χ^2^ = 6.0; *P* = .008) and HPV58 (4.7%; χ^2^ = 3.3; *P* = .044) showed significant differences in infection compared to the control group (Tables 4 and 5). In the fungal infection group, HPV39 (2.7%; χ^2^ = 4.7; *P* = .032) showed a significant difference in infection compared to the control group. In the cervical lesion group, HPV16, HPV39, HPV51, HPV52, HPV56 are all significant differences from the control group.

**Table 4:** Prevalence of the different HPV genotypes among disease.

Genotypes	Sorts									
	Health check		Pelvic		Cervical lesions		Fungal vaginitis		Bacterial vaginitis	
	No.	Ratio	No.	Ratio	No.	Ratio	No.	Ratio	No.	Ratio
HPV 16	15	3.5%	12	5.0%	63	9.6%	8	2.7%	45	4.8%
HPV 18	6	1.4%	2	0.8%	15	2.3%	6	2.0%	13	1.4%
HPV 31	4	0.9%	6	2.5%	14	2.1%	2	0.7%	10	1.1%
HPV 33	4	0.9%	3	1.3%	7	1.1%	3	1.0%	8	0.8%
HPV 35	5	1.2%	3	1.3%	15	2.3%	0	0.0%	6	0.6%
HPV 39	3	0.7%	6	2.5%	20	3.0%	8	2.7%	19	2.0%
HPV 45	1	0.2%	1	0.4%	0	0.0%	0	0.0%	5	0.5%
HPV 51	6	1.4%	4	1.7%	21	3.2%	7	2.4%	18	1.9%
HPV 52	15	3.5%	14	5.9%	64	9.7%	16	5.4%	65	6.9%
HPV 56	7	1.6%	3	1.3%	22	3.3%	6	2.0%	22	2.3%
HPV 58	11	2.6%	4	1.7%	46	7.0%	11	3.7%	44	4.7%
HPV 59	3	0.7%	2	0.8%	15	2.3%	2	0.7%	9	1.0%
HPV 66	4	0.9%	4	1.7%	28	4.2%	3	1.0%	22	2.3%
HPV 68	4	0.9%	4	1.7%	17	2.6%	3	1.0%	18	1.9%
HPV 82	3	0.7%	2	0.8%	1	0.2%	1	0.3%	4	0.4%
HPV6 + 11	2	0.5%	0	0.0%	4	0.6%	0	0.0%	1	0.1%

The ratio is calculated by the HPV-genotypes positive individuals divided the total individuals.

No.= sample number.

**Table 5:** Chi-square test the top sixth HPV genotypes among disease.

Genotypes	Disease							
	Pelvic		Cervical lesions		Fungal vaginitis		Bacterial vaginitis	
	χ2	*P*	χ2	*P*	χ2	*P*	χ2	*P*
HPV 16	0.9	.222	14.0	.000	0.4	.351	1.1	.185
HPV 18	0.4	.409	1.0	.220	0.4	.359	0.1	.568
HPV 39	3.7	.059	6.8	.006	4.7	.032	3.2	.054
HPV 51	1.2	.551	3.3	.048	0.9	.249	0.4	.343
HPV 52	2.1	.167	14.6	.000	1.5	.147	6.0	.008
HPV 56	0.1	.493	2.8	.048	0.1	.453	0.7	.275
HPV 58	0.6	.327	10.0	.001	0.8	.254	3.3	.044

All 4 groups were compared with the health check group.

## 4. Discussion

The microecology of the reproductive tract changed with age, this phenomenon is related to factors such as age-related reproductive and marital status, hormones, and lifestyle habits.^[19,20]^ In this study, the peak incidence of pelvic inflammatory disease was found 40 to 45 years old, which is a period after pregnancy and before menopause.^[21]^ High incidence of bacterial vaginitis in the 20 to 45 year group, this data is consisted with the Grenadian women, which showed high incidence of bacterial vaginitis in the 20 to 29 years.^[22]^ Pregnancy and sexual activity may explain infection during this period. The peak period for fungal vaginitis was found to be 30 to 35 years old, occurring mostly before pregnancy. This finding is consistent with a previous study by Ting Zhao, which found the highest incidence of fungal vaginitis at 20-29 years old in Yunnan provinces of Chinese.^[23]^ Cervical lesions exhibit a typical cumulative time effect, which is consistent with previous Chinese findings.

It has been discovered that HPV infection are closely related to the type and abundance of vaginal microorganisms.^[24]^ A study conducted in Northwest China found that HPV types 16 and 18 are commonly found in women with normal vaginal microecology, while HPV types 16 and 52 are common in women with vaginal infections.^[10]^ In this study, HPV types 52 and 58 showed significant differences in infection rates between bacterial group and control group, which may be due to the influence of bacterial infections on the infectivity of HPV types 52 and 58. This research found HPV 39 infection rate was significantly different between the fungal vaginitis group and the control group. Fungal vaginitis is mainly caused by *C albicans*, whether *C albicans* and HPV synergistically affect vaginal microecology requires further investigation.^[25]^ In adding, this study also found significant differences in HPV types 16, 39, 51, 52, and 56 between cervical lesions and the control group, providing new evidence for HPV vaccine administration.

Interesting, vaginal microecology can affect the process of HPV infection through various mechanisms.^[26,27]^ Vaginal lactobacilli maintain a low pH, prevent HPV invasion, and produce bacteriocins, effectively inhibiting HPV entry into basal keratinocytes of the cervix.^[28]^ Anaerobic bacteria in vaginal bacterial vaginosis produce enzymes and metabolites that may disrupt the barrier of cervical epithelial cells and promote HPV entry.^[29,30]^ In addition, colonization of *C albicans* in vaginal candidiasis can affect HPV infection by altering vaginal acidity and alkalinity.

## 5. Limitations of this study

The samples were taken from outpatient patients rather than a cohort population, which may result in bias in disease incidence compared to the general population. The overall sample size of the study was not large, especially the limited number of patients with pelvic inflammatory disease. On the data collection, upper reproductive tract infections such as and cervical lesions may be influenced by lower reproductive tract infections such as vaginal infections.^[31]^

## 6. Conclusion

This study characterized the HPV infection types and rates in common reproductive tract infections such as pelvic inflammatory disease, cervical lesions, bacterial vaginosis, and fungal vaginitis. Through scientific statistical analysis, not only the study found a correlation between HPV infection and cervical lesions, but also discovered associations between HPV infection and bacterial vaginosis and fungal vaginitis. This study provides new insights into the harmful effects of reproductive tract microecological imbalance and offers new perspectives for the prevention and treatment of HPV infections.

## Acknowledgments

The authors acknowledge all the people who participated in this work at Shandong Maternal and Child Health Hospital. We also thank all participants in the research.

## Author contributions

**Data curation:** Liang Wang, Ting-Ting Wang.

**Formal analysis:** Liang Wang, Ting-Ting Wang.

**Investigation:** Xiao-Li Qu.

**Writing – original draft:** Yu-Xia Zhou, Xiao-Qian Zhang.

**Writing – review & editing:** Yu-Xia Zhou, Xiao-Qian Zhang.
